# Engineering Unequal Antipolar Displacement in Ferromagnetic Layered Oxide Heterostructures

**DOI:** 10.1002/adma.202513458

**Published:** 2026-02-25

**Authors:** Jonathan Spring, Natalya S. Fedorova, Alexander Vogel, Javier Herrero‐Martín, Evgenios Stylianidis, Pavlo Zubko, Jorge Íñiguez‐González, Marta D. Rossell, Marta Gibert

**Affiliations:** ^1^ Physik‐Institut University of Zurich Zurich 8057 Switzerland; ^2^ Smart Materials Unit Luxembourg Institute of Science and Technology Esch/Alzette L‐4362 Luxembourg; ^3^ Electron Microscopy Center Empa–Swiss Federal Laboratories for Materials Science and Technology Dübendorf 8600 Switzerland; ^4^ Swiss Nanoscience Institute University of Basel Basel 4056 Switzerland; ^5^ ALBA Synchrotron Light Source Cerdanyola del Vallès 08290 Spain; ^6^ London Centre for Nanotechnology and Department of Physics and Astronomy University College London 17‐19 Gordon Street London WC1H 0AH UK; ^7^ Department of Physics and Materials Science University of Luxembourg Belvaux L‐4422 Luxembourg; ^8^ Institute of Solid State Physics TU Wien Vienna 1040 Austria

**Keywords:** antipolar diplacements, double perovskites, ferromagnetism, oxide heterostructures

## Abstract

Heterostructure engineering provides a versatile route for tailoring emergent functionalities that are often difficult to realize in single‐phase materials. In this work, the focus is on superlattices composed of the insulating and ferromagnetic double perovskites La_2_NiMnO_6_ and Sm_2_NiMnO_6_, which constitute an intriguing model system. These layered structures are predicted to feature unequal antipolar displacements of the La and Sm ions; when combined with odd periodicity stacking, this structural motif is expected to give rise to polar behavior. The respective superlattices are grown with atomic precision and display robust ferromagnetism, as confirmed by in‐house magnetometry and synchrotron measurements. Scanning transmission electron microscopy combined with first‐principles calculations confirms the presence of the predicted unequal antipolar displacements, paving the way for the realization of hybrid improper ferroelectricity in such oxide heterostructures.

## Graphical abstract

Atomically precise superlattices of the double perovskites La_2_NiMnO_6_ and Sm_2_NiMnO_6_ are grown to engineer an unequal antipolar displacement of the La and Sm ions. These structural motifs are confirmed by the scanning transmission electron microscopy and first‐principles calculations. Combined with their robust ferromagnetism, these heterostructures demonstrate a promising pathway toward hybrid improper ferroelectricity in artificially layered oxide systems.

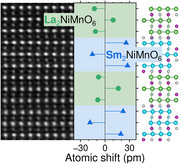

## 1 Introduction

One of the greatest challenges in solid state physics and materials science is the engineering of multiferroic systems, combining both magnetic order and ferroelectricity.^[^ ^1^, ^2^ ^]^ These highly sought‐after materials could be crucial to the development of future high‐efficiency and low‐power electronics.^[^ ^3^ ^]^ In oxides, materials displaying both properties in a given phase are rare, given that both orders tend to be mutually exclusive in perovskite oxides.^[^ ^4^ ^]^ BiFeO_3_ is one of the most prominent and most studied single‐phase multiferroics.^[^ ^5^, ^6^ ^]^ Moving away from single‐phase materials, multiferroicity can be found in oxide heterostructures grown with atomic precision.^[^ ^3^, ^7^ ^]^ Interestingly, the artificial layering of different perovskite oxide magnetic materials, combining their non‐polar rotation modes, has been suggested as a design principle to achieve a polar, magnetic heterostructure.^[^ ^8^ ^]^ In contrast to proper ferroelectricity, the electric polarization here is a secondary effect to a primary structural transition. It arises from a trilinear coupling term in the thermodynamic free energy of the form γ*PQ*_1_ *Q*_2_, where γ is the coupling parameter, *P* is the polarization and *Q*_1_ and *Q*_2_ are two non‐polar modes, such as the oxygen octahedra BO_6_ tilts and rotations in perovskite oxides. Note that neither *Q*_1_ nor *Q*_2_ alone breaks inversion symmetry; instead, it is their simultaneous occurrence that leads to a net polarization. In the case of switchable electric polarization, which requires a change in the sign of the product *Q*_1_ *Q*_2_, this mechanism is referred to as hybrid improper ferroelectricity,^[^ ^9^ ^]^ and is demonstrated in naturally layered compounds like Ca_3_Ti_2_O_7_ or Sr_3_Sn_2_O_7_.^[^ ^10^, ^11^, ^12^ ^]^. The orthorhombic ABO_3_ perovskites with space group *Pbnm* feature a characteristic tilt pattern of the BO_6_ octahedra described as *a* ^−^ *a* ^−^ *c* ^+^ in Glazer notation.^[^ ^13^, ^14^ ^]^ The simultaneous occurrence of in‐phase and antiphase octahedral tilts leads to antipolar displacements of the A‐site ions along *b*, via a trilinear coupling term analogous to the one just mentioned.^[^ ^15^ ^]^ This is also true along *a*, though to a much lesser extent. Successive perovskite layers (along *c*) show A‐site displacements in opposite directions. However, the resulting electric dipoles will compensate as they are all of equal magnitude. In contrast, in *c*‐layered (ABO_3_)_*m*_/(A'BO_3_)_*n*_ structures featuring A‐site ions of different sizes (where *m* and *n* are the thicknesses of the corresponding layers in unit cells), the antipolar displacements of A and A' are unequal in magnitude. If *n* and *m* are odd, this leads to uncompensated electric dipoles within every layer; the addition of such layer dipoles is non‐zero and independent of the layer thickness. It thus results in a macroscopic electric polarization and, if said polarization is switchable, it leads to the aforementioned hybrid improper ferroelectricity. The odd number of unit cells is necessary because, otherwise, the electric dipoles would cancel out within the individual layers. Note also that, for example, if *m* is odd and *n* is even, the electric dipoles of consecutive (ABO_3_)_*m*_ layers would cancel each other. The highest polarization values are predicted for (ABO_3_)_1_/(A'BO_3_)_1_ superlattices. Increasing the periodicity reduces the expected polarization as *P* = *P*_0_/(*n* + *m*), where *P*_0_ is a parameter proportional (except for a volume factor) to the total electric dipole originating from the addition of the mentioned layer dipoles.^[^ ^16^ ^]^ For a *m* = *n* = 3 superlattice, for example, the polarization will decrease to ∼1/3 of the value of the *m* = *n* = 1 one.

**Figure 1:**
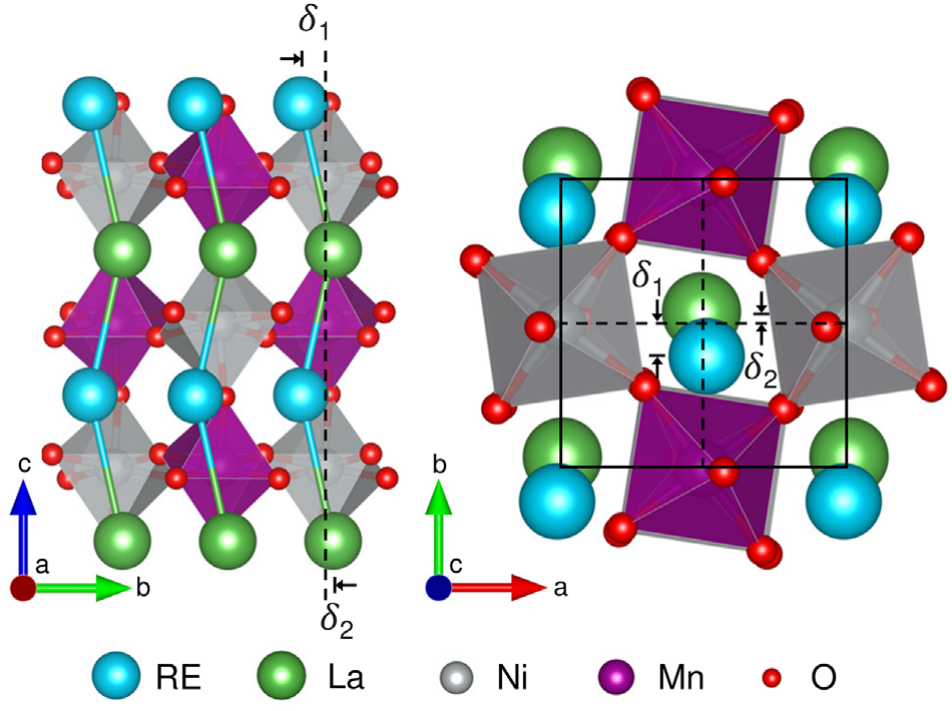
Illustration of a La_2_NiMnO_6_/RE_2_NiMnO_6_ (RE ≠ La) (1,1) superlattice projected along the orthorhombic *a* (left) and *c* (right) axis. The in‐plane antipolar displacement of the RE and La ions are labeled as δ_1_ and δ_2_, respectively. The unequal magnitude of δ_1_ and δ_2_ in combination with the odd superlattice periodicity is required for the emergence of a net electric polarization along *b*.

**Figure** **1** illustrates the dissimilar antipolar distortions necessary to induce a net electric dipole in such artificially layered heterostructures. The double perovskites RE_2_NiMnO_6_ (RE = rare earth) are used as a model system. These double‐perovskite building blocks feature a rock salt ordering of the B‐site Ni/Mn ions, resulting in a distinct electronic configuration of Ni^2 +^ and Mn^4 +^, which leads to ferromagnetic superexchange.^[^ ^17^, ^18^ ^]^ The Curie temperature (*T*_C_) of La_2_NiMnO_6_ is 280 K, and for the other members of the family it decreases linearly with the ionic radius of the RE.^[^ ^19^ ^]^ First‐principles calculations predict emerging hybrid improper ferroelectricity in odd‐period ferromagnetic (La_2_NiMnO_6_)_*m*_/(RE_2_NiMnO_6_)_*n*_ (RE ≠ La) superlattices, with the electric polarization and Curie temperature expected to decrease with the size of the chosen RE.^[^ ^20^ ^]^

In this work, we grow atomically precise La_2_NiMnO_6_/Sm_2_NiMnO_6_ (LNMO/SNMO) superlattices by sputtering with in situ reflection high‐energy electron diffraction (RHEED). In‐house magnetometry combined with synchrotron experiments confirms robust ferromagnetism. Appropriate substrate choice allows us to induce the desired superlattice orientation. Finally, a detailed electron microscopy study reveals a pronounced antipolar displacement with unequal magnitude in the LNMO and SNMO layers, in essential agreement with our first‐principles calculations. This is key to the development of electric polarization and constitutes an important step toward establishing hybrid improper ferroelectricity in layered oxide heterostructures. It further exemplifies how superlattice engineering allows us to accurately manipulate their structural and functional properties.

## 2 Results and Discussion

### 2.1 Superlattice Growth and Structural Characterization

**Figure 2:**
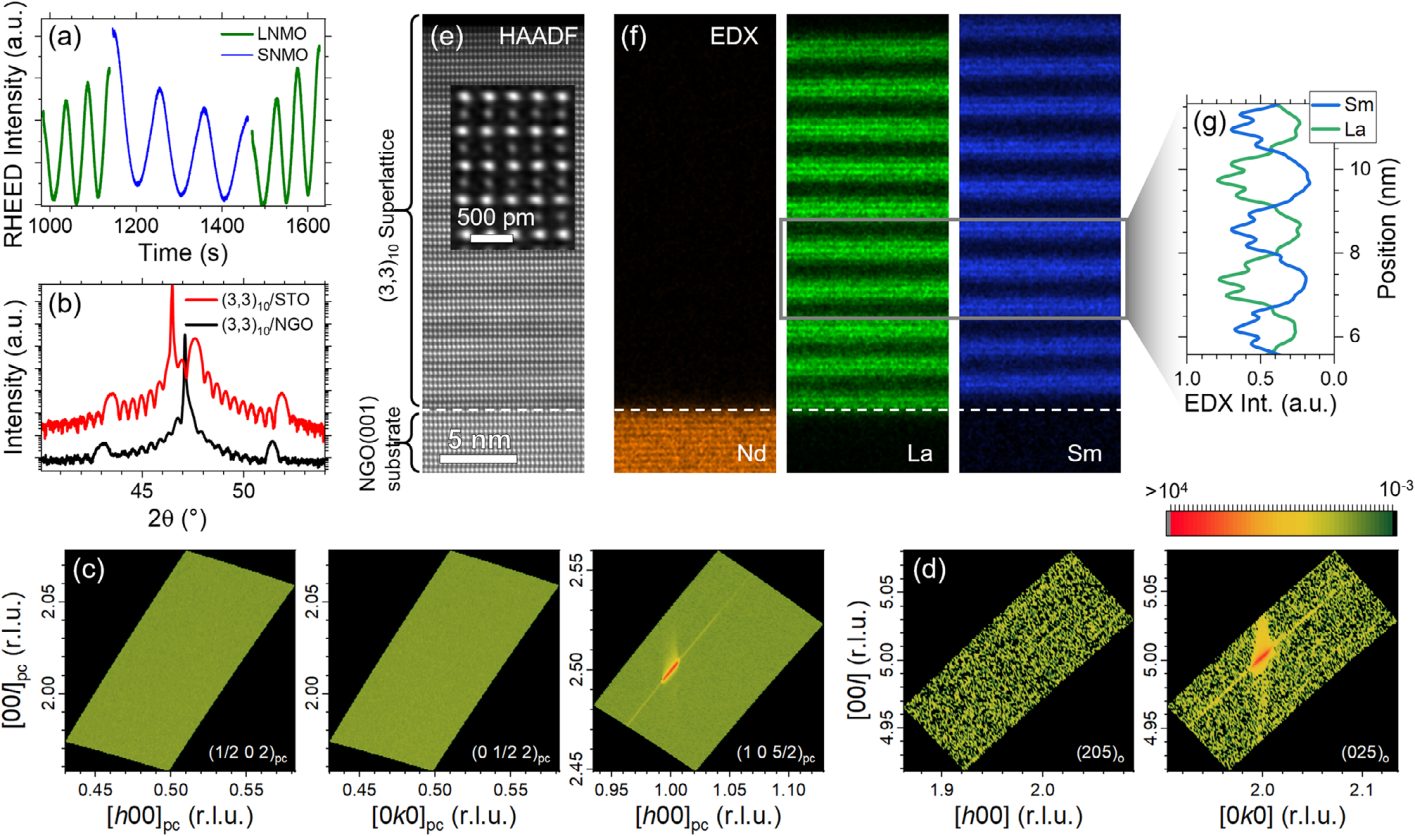
Structural characterization. a) Tracking of the RHEED intensity during the sputter deposition of a (3, 3)_10_ superlattice on NGO(001). b) XRD for (3, 3)_10_ superlattices grown on NGO (black) and STO (red). c) RSMs around the pseudocubic (1/2 0 2)_pc_, (0 1/2 2)_pc_, and (1 0 5/2)_pc_ positions indicate an out‐of‐plane orientation of both the NGO substrate's and the superlattice's *c*‐axis. d) RSMs around the orthorhombic (2 0 5)_o_ and (0 2 5)_o_ positions indicate only one in‐plane orientation of the superlattice grown on NGO. The horizontal color bar represents the intensity scale common to all RSM plots. e–g) Cross‐sectional STEM imaging of a (3, 3)_10_ superlattice along the orthorhombic [100] direction of the NGO(001) substrate. The HAADF‐STEM data in (e) highlights the in‐plane shifts of the A‐site ions (zig‐zag pattern in the out‐of‐plane direction) corresponding to the (001) orientation of the superlattice with *a* ^−^ *a* ^−^ *c* ^+^ tilt pattern. The EDX data in (f,g) demonstrates the (3,3) superlattice layering.

The LNMO/SNMO superlattices are grown on a single crystal orthorhombic NdGaO_3_(001) (NGO) and cubic SrTiO_3_(001) (STO) substrates. Using the pseudocubic (pc) lattice parameters of 3.881 and 3.823 Å for LNMO and SNMO,^[^ ^19^ ^]^ respectively, this leads to strain values of −0.54 % (+0.62 %) in the LNMO layers and +0.97 % (+2.14 %) in the SNMO layers for samples grown on NGO (STO). The LNMO/SNMO superlattices are grown in different periodicities, denoted as (*x*, *x*)_*y*_, where *x* is the number of pc unit cells per layer of LNMO and SNMO, respectively, and *y* is the number of repetitions of the LNMO/SNMO double layer. The notation (x,x) is used when referring to the periodically repeated superlattices considered in our DFT simulations. *x* and *y* are chosen such that the total thickness is 60 pc unit cells. Here, we will mainly focus on the properties of the (3, 3)_10_ periodicity. Our custom‐built sputtering system equipped with RHEED allows us to control the superlattice periodicity in situ. By switching the deposition between the two constituents, LNMO and SNMO, at exactly the maximum of a RHEED intensity oscillation, **Figure** **2**a, we optimize the sharpness of the superlattice interfaces. This leads to high‐quality, epitaxially strained heterostructures, as exemplified by X‐ray diffraction (XRD) around the (002)_*pc*_ substrate peak for (3, 3)_10_ superlattices grown on NGO and STO in Figure 2b. Distinct Laue oscillations and the observation of superlattice satellite peaks attest to the high crystal quality of the heterostructures. For the sample grown on the NGO, the main diffraction peak of the superlattice lies only at slightly higher values of 2θ than the substrate peak, and hence both peaks are heavily convoluted. Additional XRD data are shown in Figure S1 (Supporting Information). We also present atomic force microscopy (AFM) topography for samples on both substrates in Figure S2 (Supporting Information), revealing a smooth step‐and‐terrace structure.

In order to engineer the desired unequal in‐plane antipolar displacement of the La and Sm ions, control of the superlattice orientation is crucial. As illustrated in Figure 1, the A‐site displacement occurs mainly along *b*. For potential hybrid improper ferroelectricity, this has to correspond to an in‐plane direction of the superlattice, and the out‐of‐plane layering has to occur along *c*. This corresponds to a rotation pattern of *a* ^−^ *a* ^−^ *c* ^+^. As the orthorhombic NGO(001) substrates naturally display this rotation pattern, they are an a priori choice for achieving the desired growth orientation. To verify the superlattice orientation, Figure 2c shows reciprocal space maps (RSMs) around (1/2 0 2)_pc_, (0 1/2 2)_pc_ and (1 0 5/2)_pc_. As the substrate has its orthorhombic *c*‐axis in the out‐of‐plane direction, we find an intense substrate peak at (1 0 5/2)_pc_ originating from the doubling of the pseudocubic unit cell. As no peaks arise in the other two directions, we conclude that the superlattice *c*‐axis is following the substrate orientation, i.e., it is also oriented in the out‐of‐plane direction. The superlattice peak is expected to be very close to the substrate peak, c.f. panel (b). Hence, in the RSM, it is hidden in the substrate peak, and only a slight streaking at *l* values larger than the substrate is observed, originating from the superlattice. To determine the in‐plane orientation, we present RSMs around the orthorhombic (2 0 5) and (0 2 5) substrate positions in Figure 2d. For structures with *Pbnm* symmetry, the (2 0 5) diffraction condition is forbidden, while (0 2 5) is allowed.^[^ ^21^ ^]^ Hence, we observe an intense substrate diffraction peak only at (0 2 5) and not at (2 0 5). The absence of any signal around (2 0 5) means that the superlattice entirely follows the substrate's in‐plane orientation. Again, for the (0 2 5) diffraction condition, the streaking at *l* values larger than the substrate likely originates from the superlattice. To conclude the RSM analysis, the superlattice has its orthorhombic *c*‐axis oriented out‐of‐plane, and there exists only one in‐plane orientation of the orthorhombic *a*‐ and *b*‐axis. This understanding of the superlattice orientation will be significant for cutting the lamella for electron microscopy in the desired orientation. For the samples grown on STO(001), we also find the superlattice *c*‐axis in the out‐of‐plane direction. However, in the in‐plane direction, two orientational domains occur with the superlattice *a*‐ and *b*‐axis along both of the equivalent [100] and [010] directions of the cubic substrate, Figure S3 (Supporting Information).

The structure of the (3, 3)_10_ superlattice grown on NGO is further characterized by cross‐sectional scanning transmission electron microscopy (STEM) along the orthorhombic [100] direction of the NGO(001) substrate, Figure 2e–g. This corresponds to [110]_pc_. The high‐angle annular dark‐field (HAADF)‐STEM image in panel (e) exemplifies the structural quality of our superlattices and further reveals a zig‐zag pattern in the sample's out‐of‐plane direction. This confirms the desired (001) orientation of the superlattice. The energy‐dispersive X‐ray (EDX) elemental maps in panel (f) illustrate the distinct (3,3) layering of the superlattice. Extracting the EDX intensity profiles for La and Sm in panel (g) shows that the interfacial intermixing is kept below one pc unit cell. EDX data for the remaining elements is shown in Figure S4 (Supporting Information). Regarding the electronic structure of the B‐site Ni/Mn sublattice, X‐ray absorption spectroscopy (XAS) measurements reveal a predominant valence of Ni^2 +^ and Mn^4 +^ independent of superlattice periodicity and substrate used, Figure S5 (Supporting Information). This is indicative of a high degree of order between Ni and Mn, which is a prerequisite for the emergence of ferromagnetism.^[^ ^17^, ^18^, ^22^ ^]^

### 2.2 Magnetic Characterization

**Figure 3:**
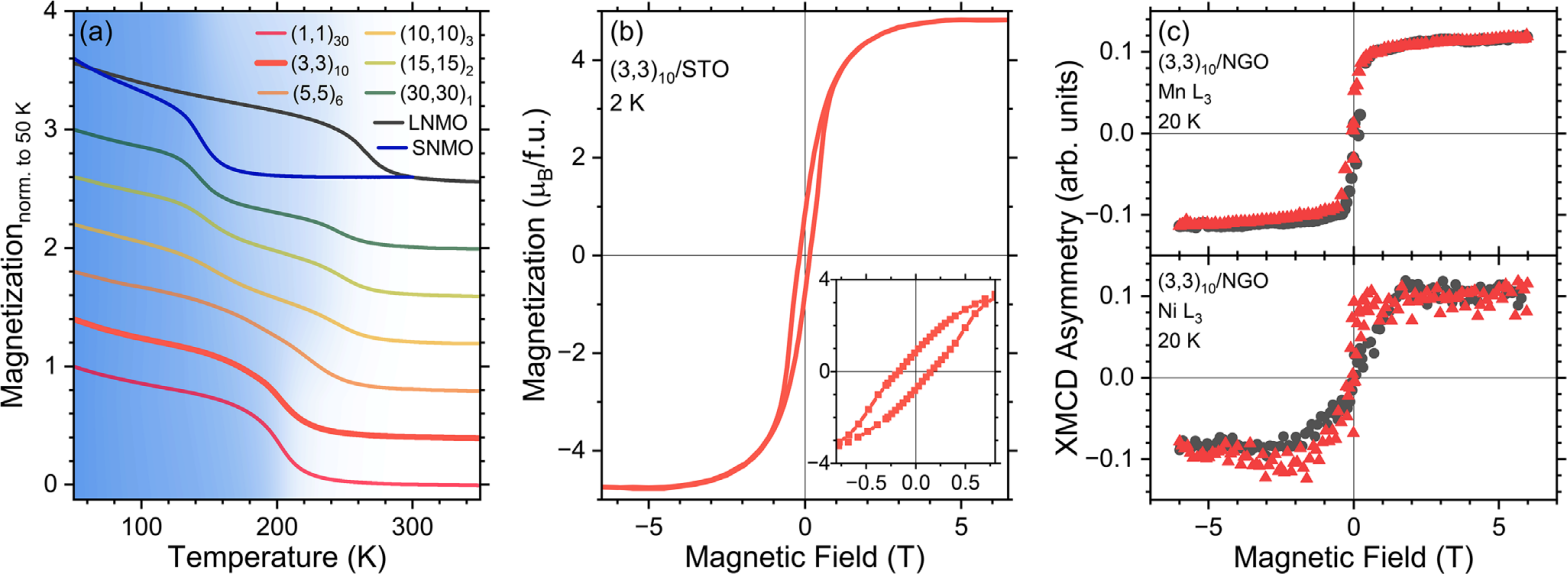
Magnetic Characterization. a) *M*(*T*) for superlattices of different periodicities on STO(001) measured at an in‐plane applied magnetic field of 0.5 T. For comparison, the M(T) curves for bare 30 pc unit cells LNMO and SNMO films are included. For the high periodicity superlattices, the two individual transitions of LNMO and SNMO are conserved, whereas they collapse into a single transition for the low periodicity samples. The curves are normalized to their value at 50 K and vertically offset for better visibility. b) *M*(*H*) for a (3, 3)_10_ superlattice on STO(001) measured at a temperature of 2 K and the magnetic field applied in the in‐plane direction. The inset shows a magnification around the origin. c) XMCD asymmetry loops for a (3, 3)_10_ superlattice on NGO(001) recorded at the Mn *L*_3_ (top) and the Ni *L*_3_ (bottom) absorption edge at a temperature of 20 K. The red triangles (black dots) correspond to measurements acquired while increasing (decreasing) the applied magnetic field.

Magnetic ordering is one of the key ingredients for multiferroicity. **Figure** **3**a shows magnetization vs temperature *M*(*T*) data for a (3, 3)_10_ superlattice together with a few other selected periodicities investigated via magnetometry. Additionally, we show data for two bare 30 pc unit cells, LNMO and SNMO thin films. As NGO substrates contribute a very large paramagnetic signal, by far overshadowing the superlattice contribution, we present the measurements for superlattices and films on STO substrates. The low periodicity (3, 3)_10_ and (1, 1)_30_ samples feature a *T*_C_ of ≈225 K. This is very close to the value of 223 K for a (1,1) superlattice predicted using density functional theory (DFT).^[^ ^20^ ^]^ It is also close to the average (219 K) of the *T*_C_s reported for bulk LNMO (280 K) and SNMO (157 K), respectively.^[^ ^19^ ^]^ Turning to the higher periodicities in Figure 3a, the transition separates into two individual transitions belonging to the LNMO and SNMO layers, respectively. For the (30, 30)_1_ sample, we find two distinct magnetic transitions very close to the individual *T*_C_s of LNMO and SNMO. The propagation of the magnetic order parameter across superlattice interfaces causes the individual LNMO and SNMO transitions to merge into a single transition at low periodicities.^[^ ^23^ ^]^

Looking at the magnetization vs applied magnetic field *M*(*H*), we confirm the ferromagnetic behavior of the LNMO/SNMO superlattices. For the (3, 3)_10_ superlattice at 2 K, Figure 3b, the *M*(*H*) loop is characterized by a saturation magnetization of 4.7 μ_B_ per formula unit of (L/S)NMO (f.u.) and a remnant magnetization of 0.8 μ_B_/f.u. To verify if this is also true for the sample grown on NGO(001), we employ element‐specific X‐ray magnetic circular dichroism (XMCD). Figure 3c shows XMCD asymmetry loops as a function of the applied magnetic field recorded at the Mn *L*_3_ (top) and Ni *L*_3_ (bottom) absorption edges. The respective XMCD energy spectra are presented in Figure S6 (Supporting Information). The Ni and Mn sublattices both display ferromagnetic behavior with saturating magnetization at high applied fields. The same behavior is observed for Ni and Mn on the (3, 3)_10_ superlattice on STO(001), Figure S7 (Supporting Information), as expected from the magnetometry data in Figure 3a,b.

### 2.3 Engineering the Unequal Antipolar Displacement

**Figure 4:**
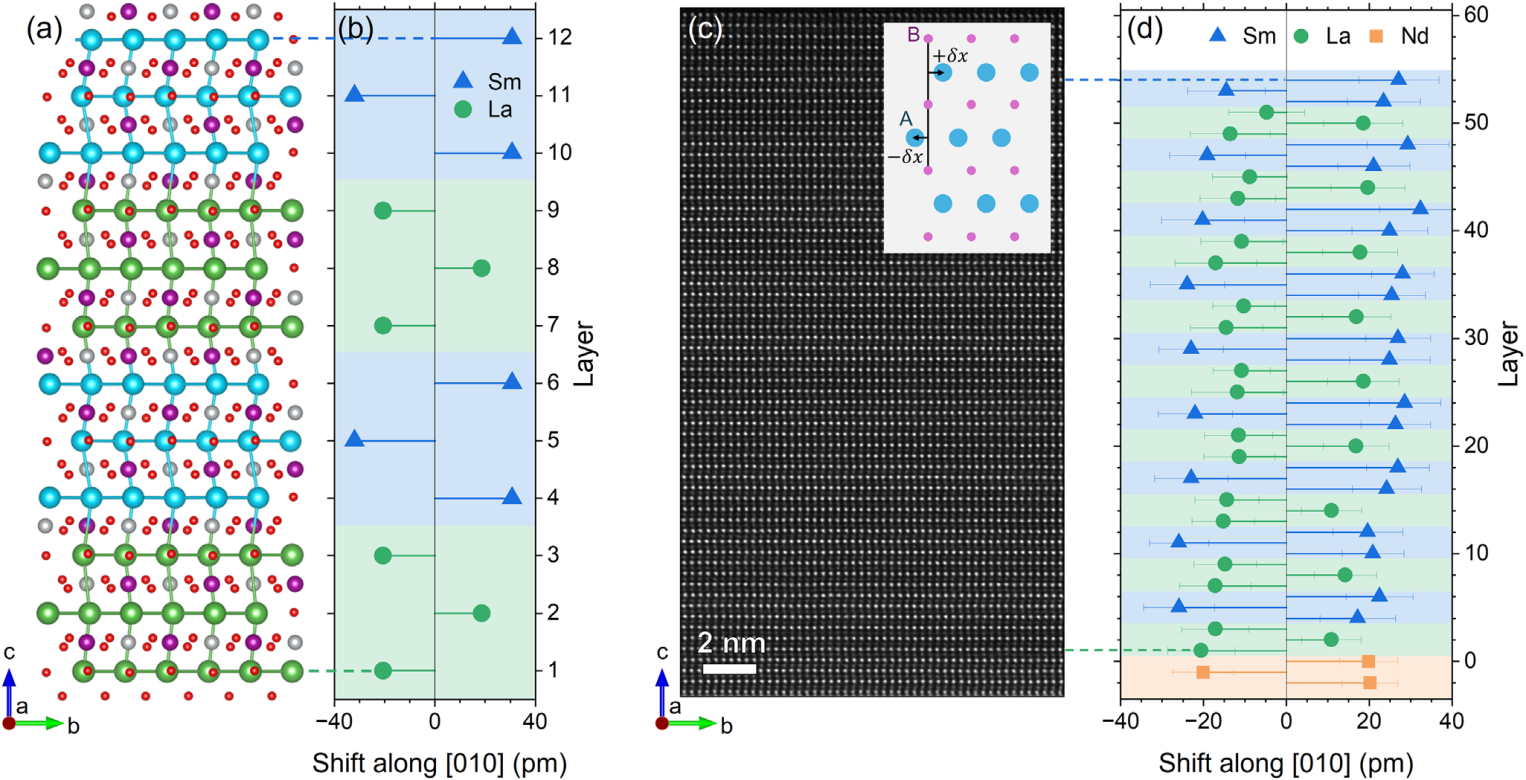
Characterization of the antipolar displacement. a) DFT‐calculated structure of a (3,3) superlattice projected along *a*. The La ions are shown in green, Sm in blue, Ni in grey, Mn in purple and O in red. b) Displacements of the La and Sm ions along the *b* axis extracted from the structure in (a). c) HAADF‐STEM image of a (3, 3)_10_ superlattice imaged along the [100] axis. The inset illustrates how the A‐site displacements (±δ_*x*_) are determined. d) Layer‐averaged displacements of the Sm and La ions with respect to the position of the B‐site ions in the layers above and below. The error bars correspond to the standard error of the mean within one layer. The data is shifted horizontally such that the displacement is symmetric around 0 for the Nd ions in the substrate.

As we have shown in the previous sections, our LNMO/SNMO superlattices display excellent structural quality with the *c*‐axis in the out‐of‐plane direction and robust ferromagnetism. To characterize the extent of the antipolar displacement of the La and Sm ions, we employ a combination of first‐principles calculations and electron microscopy. As a point of reference, the structure of a (3,3) superlattice with its in‐plane *a* and *b* lattice parameters clamped to the values of NGO(001) is calculated using DFT, **Figure** **4**a. The structure is projected along its *a* axis, highlighting the antipolar displacement along *b*. In Figure 4b, we extract the displacements of the La and Sm ions from their ideal cubic positions resolved by layer. We find average displacements of 20.0 and 31.2 pm for La and Sm, respectively. Using the Berry‐phase approach,^[^ ^24^ ^]^ we find an electric polarization value of $2.20\;\mu Ccm^{-2}$. For comparison, we also calculated using DFT the polarization for a (1,1) superlattice as $5.49\;\mu Ccm^{-2}$, which is close to the previously published value of $\approx 5.9\;\mu Ccm^{-2}$.^[^ ^20^ ^]^ Note that the data published in [20] is for unstrained superlattices. The ratio of the DFT‐predicted polarization for a (3,3) to a (1,1) superlattice is ≈1/3, in agreement with the phenomenological and symmetry‐based arguments in Ref. [16].

Next, we employ HAADF‐STEM to access the A‐site displacements in our (3, 3)_10_ LNMO/SNMO superlattice grown on NGO(001). The lamella is cut parallel to the substrate's *bc*‐plane and imaged along *a*, Figure 4c. For each A‐ion (La and Sm), we measure the displacement along *b* with respect to the position of the B‐ions (Ni and Mn) above and below. This is illustrated in the inset of Figure 4c. The resulting displacement values are averaged in each layer and displayed in Figure 4d. The top LNMO/SNMO double layer is excluded from this analysis as the values close to the surface are unreliable. We can clearly identify the presence of unequal antipolar displacement. This is exemplified in the distinctly visible (3,3) periodicity, with three successive layers showing smaller (larger) atomic displacements corresponding to the LNMO (SNMO) layers. We note that the displacements are not exactly symmetric around 0. This is most likely the result of a slight tilt of the superlattice with respect to the substrate, and it prohibits determining the absolute displacements. Instead, we choose to look at the average La–La and Sm–Sm distances within one LNMO or SNMO slab. Averaged over five different STEM images, we find values of 25.2 ± 6.7 and 42.9 ± 6.7 pm for the La–La and Sm–Sm distances, respectively, highlighting the presence of unequal antipolar displacement. The values for the individual STEM images are presented in Table S1 (Supporting Information). These experimental values are somewhat smaller than what we find for our DFT‐calculated structure, where the respective distances are 39.3 pm (La–La) and 62.7 pm (Sm–Sm). Probably, this quantitative difference reflects the fact that the DFT calculations correspond to the limit of very low temperatures (nominally, 0 K), where the structural distortions are fully developed, while our STEM images are acquired at room temperature. At any rate, looking at the ratio Sm–Sm/La–La, we find an experimental value of 1.71 ± 0.17, very close to the theoretical value of 1.60.

Assuming that the measured antipolar displacement occurs symmetrically around the ideal cubic A‐positions and that the electric dipoles depend exclusively and linearly on the displacements of the A‐ions (nominal charge of 3+) along *b*, we can estimate a potential polarization value of $1.24\pm 0.67\;\mu Ccm^{-2}$ for our (3, 3)_10_ superlattice. Applying the same rationale to the displacements in our DFT‐predicted (3,3) structure (calculated at 0 K) results in a polarization of $1.57\;\mu Ccm^{-2}$. Of course, these values are heavily dependent on the stated assumptions, as can be seen by comparison to the DFT value using the Berry‐phase approach ($2.20\;\mu Ccm^{-2}$). Nevertheless, they strongly suggest that the theoretical and experimental structures are in good agreement, and we have, in fact, realized the desired unequal antipolar displacement in our superlattices. Finally, we note that initial attempts to switch the electric polarization by applying an in‐plane electric field through interdigitated top electrodes have unfortunately not been successful so far. Switching the polarization would require the reversal of the octahedral tilting pattern, which may constitute a sizable energy barrier (see Figure S9 and Table S2, Supporting Information). This could potentially be reduced through suitable doping of the system. In addition, the somewhat leaky nature of the samples might also impede the polarization switching. While we clearly demonstrate unequal antipolar displacement, suggesting the presence of electric polarization, further investigation toward the goal of polarization switching, and hence hybrid improper ferroelectricity, will be required.

## 3 Conclusion

In this work, we showed that atomically precise heterostructures of the double perovskites La_2_NiMnO_6_ and Sm_2_NiMnO_6_ display a combination of robust ferromagnetism and unequal antipolar displacement of the La and Sm ions. In combination with the odd periodicity layering of our superlattices and supported by first‐principles calculations, this constitutes an important step toward establishing hybrid improper ferroelectricity in artificially layered oxide systems. Combined with the established ferromagnetism, our La_2_NiMnO_6_/Sm_2_NiMnO_6_ superlattices provide a foundation for exploring multiferroicity in artificially layered oxides.

## 4 Experimental Section

### Superlattice Growth and Structural Characterization

The LNMO/SNMO superlattices were grown by radio‐frequency (RF) magnetron sputtering using a custom‐built system featuring in situ reflection high‐energy electron diffraction (RHEED). The integration of RHEED allowed controlling the superlattice periodicity in real time, as each RHEED intensity oscillation corresponds to the deposition of one pc unit cell of LNMO or SNMO. Deposition takes place at an RF power of 35 W and a substrate temperature of 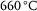 in a constant stream of Ar (35 sccm) and O_2_ (10 sccm) at a total pressure of 0.1 mbar. After deposition, the sample was cooled to room temperature in the growth atmosphere. The structural quality of the samples was characterized via X‐ray diffraction (XRD) on a Rigaku Smartlab using monochromatic Cu *K*α_1_ radiation (λ=1.5406Å). The surface quality was investigated by atomic force microscopy (AFM) using both a Park NX10 and a Nanosurf Flex.

### Magnetic Characterization

The magnetic properties of the superlattices were investigated using a Quantum Design Physical Properties Measurement System (PPMS) equipped with the Vibrating Sample Magnetometer (VSM) option. The magnetic field was applied in the plane of the film. For the *M*(*T*) measurements, a substrate curve averaged from 15 different STO substrates was subtracted. For the *M*(*H*) measurements, the diamagnetic contribution of the substrate was removed via a linear fit to the high‐field region.

### Synchrotron Measurements

X‐ray absorption (XAS) and X‐ray magnetic circular dichroism (XMCD) measurements were performed at the BOREAS beamline at the ALBA synchrotron in Barcelona, Spain.^[^ ^25^ ^]^ All measurements were acquired in grazing incidence (60 ° to the sample normal) using total electron yield detection. At 300 K, the XAS signal was measured with linear horizontal polarized X‐rays. At 20 K, it was the average between the absorption data obtained with circularly left‐ and right‐hand polarized X‐rays. The Mn and Ni reference XAS spectra were obtained on the following powder samples: MnCl_2_ (Mn^2 +^), Mn_2_O_3_ (Mn^3 +^), SrMnO_3_ (Mn^4 +^), NiO (Ni^2 +^), and NdNiO_3_ (Ni^3 +^). The XMCD signal was defined as the difference between both circular polarizations, and it was normalized by the respective XAS signal. The XMCD asymmetry loops were obtained by measuring the XMCD signal alternatingly at an on‐ and off‐resonance energy while sweeping the applied magnetic field. If necessary, one of the two branches was multiplied by a linear function to align them in the saturated regime at high applied fields.

### Scanning Transmission Electron Microscopy

Electron‐transparent samples for scanning transmission electron microscopy (STEM) investigations were prepared in cross‐section with an FEI Helios 660 G3 UC focused ion beam (FIB) operated at acceleration voltages of 30 and 5 kV after deposition of C and Pt protective layers. High‐angle annular dark‐field scanning transmission electron microscopy (HAADF‐STEM) and energy dispersive X‐ray (EDX) spectroscopy were carried out using a probe‐corrected FEI Titan Themis microscope equipped with ChemiSTEM technology. The microscope was operated at an accelerating voltage of 300 kV, probe convergence semi‐angle of 18 mrad, and collecting semi‐angles of 66 mrad to 200 mrad. The EDX spectrum image was acquired with a probe current of 814 pA and a dwell time of 5μs.

HAADF‐STEM images were obtained along the [100] direction by averaging 12‐frame time series (2048 × 2048 pixels with a dwell time of 1μs) after rigid and nonrigid registrations performed using the Smart Align software^[^ ^26^ ^]^ to correct for scan distortions. The obtained averaged images were subsequently Fourier space filtered using a bandpass filter, and the atomic column fitting was performed using the Python library Atomap^[^ ^27^ ^]^ after applying a Python‐based probe deconvolution algorithm on the HAADF‐STEM images.

As can be seen in Figure 4, the A and B atomic columns were aligned along the out‐of‐plane [001] direction of the (3,3) superlattice. Thus, here, it was determined the displacement vectors by measuring the displacement of the A cations in the image plane relative to the center of their neighboring B cations located immediately above and below. The EDX elemental maps were calculated from the EDX spectrum image using the Mn *K*α, Ni *K*α, Ga *K*α, La *L*α, Nd *L*α, and Sm *L*α lines.

### DFT Calculations

First‐principles calculations were performed using the implementation of density functional theory^[^ ^28^, ^29^ ^]^ in the Vienna ab initio Simulation Package (VASP)^[^ ^30^ ^]^ and the projector‐augmented plane wave method to treat ionic cores.^[^ ^30^, ^31^ ^]^ The generalized gradient approximation for the exchange‐correlation functional in the form of Perdew, Burke, and Ernzerhof optimized for solids (PBEsol) was utilized.^[^ ^32^ ^]^ Hubbard *U* correction (in Dudarev's scheme)^[^ ^33^ ^]^ of 3eV was applied for better treatment of Mn and Ni 3*d* electrons. The *f*‐electrons of Sm atoms were treated as core electrons. A plane‐wave basis set with an energy cutoff of 500eV was used. (1,1) and (3,3) superlattices were simulated using the 20‐ and 60‐atom cells, respectively, as shown in Figure S8 (Supporting Information). Γ‐centered 6 × 6 × 4 and 5 × 5 × 1 Monkhorst‐Pack *k*‐point grids were employed for reciprocal space integrals in the Brillouin zone corresponding to 20‐ and 60‐atom cells, respectively. Superlattices grown on (001)‐oriented NGO substrates were considered, with the *c* axis of the superlattices perpendicular to the substrate surface. The effects of strain were simulated by fixing the *a* and *b* lattice constants of the superlattices to those of the substrate (a=5.428Å and b=5.498Å). The *c* lattice constant, as well as the internal coordinates, were allowed to relax in the structural optimizations until the forces acting on the atoms were below $0.01\;eV{Å}^{-1}$. In all simulations, ferromagnetic orientation of Mn and Ni spins was imposed. The electric polarizations were computed using the Berry phase approach.^[^ ^24^ ^]^ The reference structure for computing the electric polarization for (1,1) superlattice was constructed as follows: a 20‐atom supercell (sc) was built starting from the 5‐atom cubic perovskite structure (e.g., SrTiO_3_) such that $a_{\text{sc}}=b_{\text{sc}}=a\sqrt{2}$, *c*_sc_ = 2*a* (where *a* was the lattice constant of the 5‐atom cubic cell); the atoms were then replaced so they match the atomic arrangement of the (1,1) LNMO/SNMO superlattice. Next, *a*_sc_, *b*_sc_, and *c*_sc_ were set equal to those of the relaxed (1,1) superlattice while keeping the atoms in the relative positions corresponding to the perfect cubic structure. Finally, the Berry phase approach was used to compute the change in polarization as the system was evolved from this reference configuration to the relaxed superlattice structure. A similar procedure was applied to construct the reference phase for the (3,3) superlattice. The cubic‐like phases for (1,1) and (3,3) superlattices, whose energies were used in computing the critical temperatures below which the octahedral tilts develop in these materials, were constructed in a similar way as the reference phases for computing the electric polarization. The *c* lattice constant and the internal coordinates of these structures, however, were relaxed while *a* and *b* lattice constants kept fixed to those of the NGO substrate.

## Conflict of Interest

The authors declare no conflict of interest.

## Supporting information

Supporting Information

## Data Availability

The data that support the findings of this study are available from the corresponding author upon reasonable request.
