# Supplementary material for: Engineering Unequal Antipolar Displacement in Ferromagnetic Layered Oxide Heterostructures
Source: Adv Mater. 2026 Feb 25;38(17):e13458. doi: 10.1002/adma.202513458 (PMC13003901; doi:10.1002/adma.202513458)
Supplement: Supplementary file 1 — Supporting Information [file ADMA-38-e13458-s001.pdf]

Engineering Unequal Antipolar Displacement in Ferromagnetic Layered Oxide Heterostructures – Supporting Information

Jonathan Spring Natalya Fedorova Alexander Vogel Javier Herrero-Martín Evgenios Stylianidis Pavlo Zubko Jorge Íñiguez-González Marta D. Rossell Marta Gibert**

Dr. J. Spring
Physik-Institut, University of Zurich, 8057 Zurich, Switzerland
Email Address: jonahan.spring@uzh.ch
Dr. N. Fedorova, Prof. J. Íñiguez-González
Smart Materials Unit, Luxembourg Institute of Science and Technology, L-4362, Esch/Alzette, Luxembourg
Prof. J. Íñiguez-González
Department of Physics and Materials Science, University of Luxembourg, L-4422 Belvaux, Luxembourg
Dr. A. Vogel, Dr. M. D. Rossell
Electron Microscopy Center, Empa–Swiss Federal Laboratories for Materials Science and Technology, 8600 Dübendorf, Switzerland
Dr. A. Vogel
Swiss Nanoscience Institute, University of Basel, 4056 Basel, Switzerland
Dr. J. Herrero-Martín
ALBA Synchrotron Light Source, Cerdanyola del Vallès 08290, Spain
Dr. E. Stylianidis, Prof. P. Zubko
London Centre for Nanotechnology and Department of Physics and Astronomy, University College London, London, UK
Prof. M. Gibert
Institute of Solid State Physics, TU Wien, 1040 Vienna, Austria

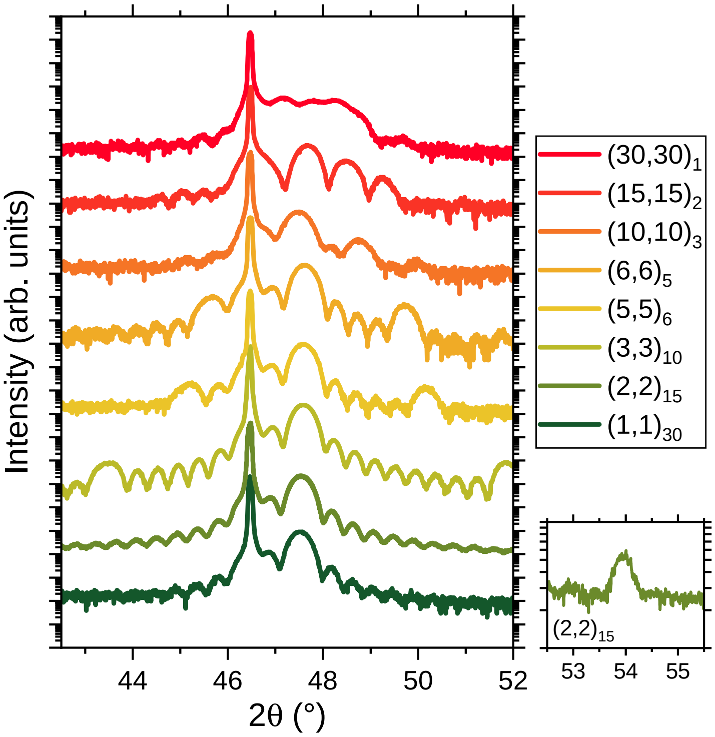

Figure S1: XRD around the STO (002) diffraction peak for LNMO/SNMO superlattices with different periodicities. The superlattice satellite peak for the $(2, 2)_{15}$ sample is shown separately as it lies outside the 2θ range of the main graph.

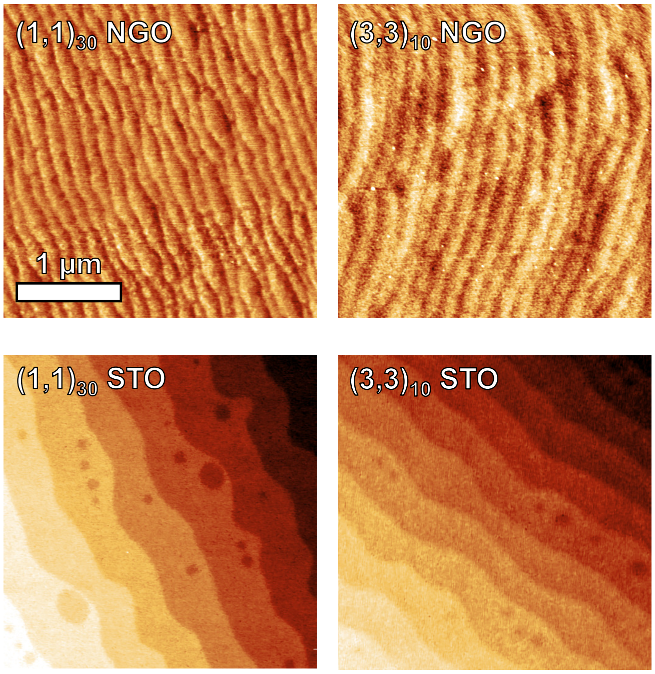

Figure S2: AFM topography for $(1, 1)_3$ and $(3, 3)_{10}$ superlattices grown on NGO(001) and STO(001) substrates.

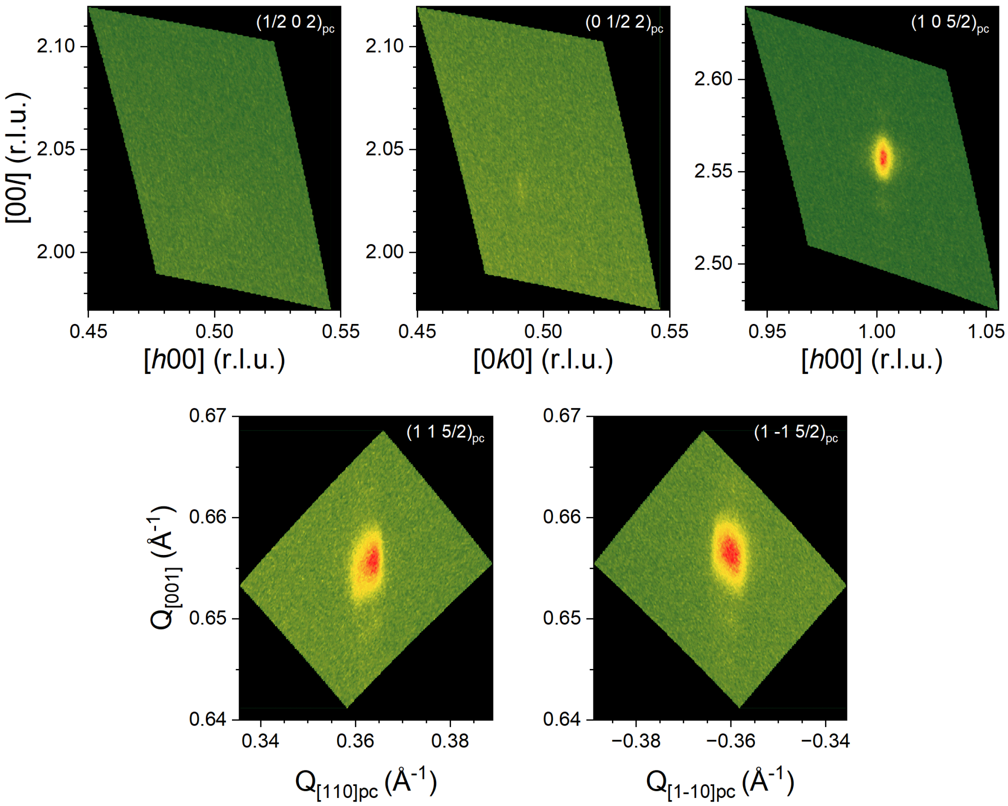

Figure S3: Reciprocal space map (RSM) analysis for a $(3,3)_{10}$ superlattice grown on STO(001). Top row: RSMs around the superlattice's $(^1/_2\ 0\ 2)_{\rm pc}$, $(0\ ^1/_2\ 2)_{\rm pc}$, and $(1\ 0\ ^5/_2)_{\rm pc}$ diffraction conditions. A doubling of the pc unit cell in the $[001]_{\rm pc}$ direction indicates the out-of-plane orientation of the orthorhombic c-axis. Bottom row: RSM around $(1\ 1\ ^5/_2)_{\rm pc}$ and $(1\ \text{-1}\ ^5/_2)_{\rm pc}$ reveals two in-plane orientations of the superlattice's orthorhombic a and b axis.

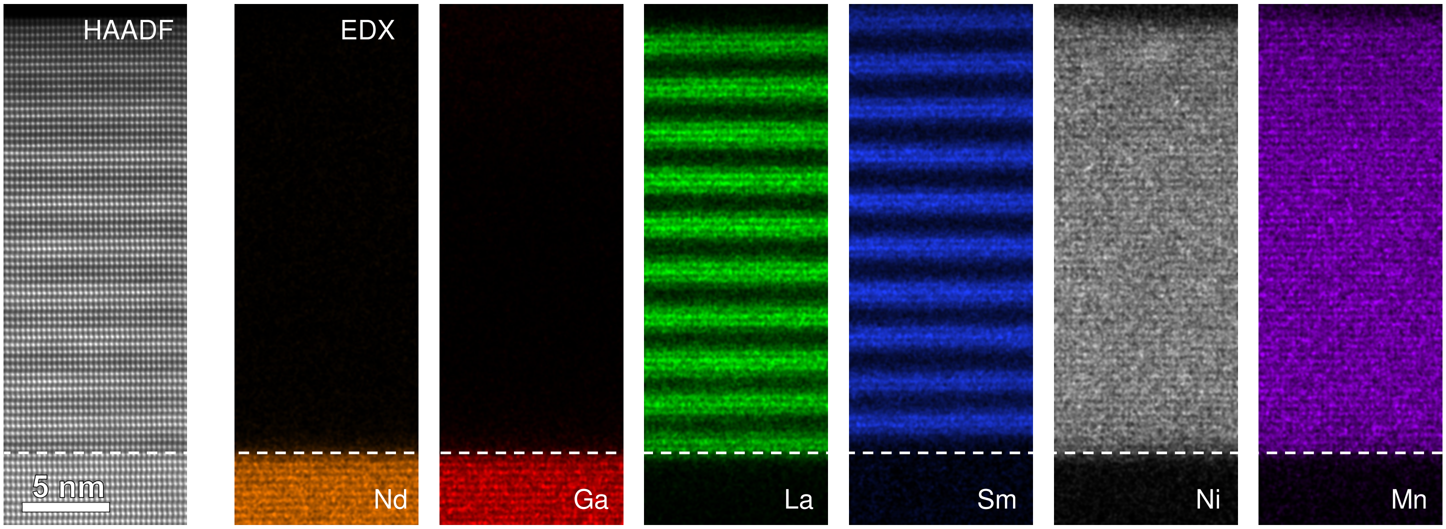

Figure S4: HAADF-STEM and EDX data for a $(3,3)_{10}$ superlattices grown on NGO(001) imaged along [100].

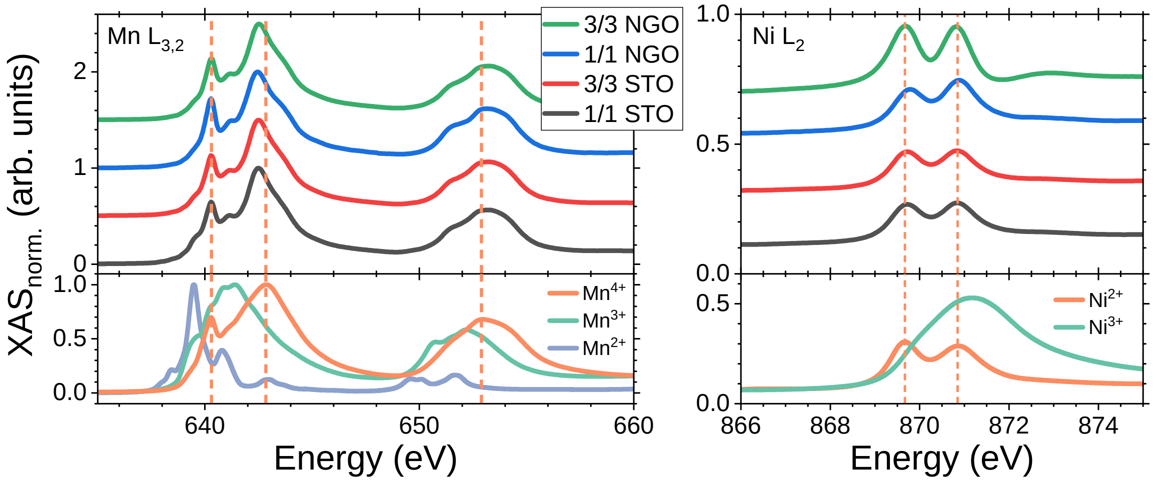

Figure S5: XAS for $(3,3)_{10}$ and $(1,1)_{30}$ superlattices on NGO(001) and STO(001) recorded at the Mn $L_{3,2}$ (left) and Ni L_2 (right) absorption edges. Comparison to the reference spectra in the bottom panels reveals a predominant valence state of Ni^{2+} and Mn^{4+} in all samples. For Mn, the measurements were performed at 300 K (except the 1/1 STO sample at 20 K). For Ni, the measurements were performed at 20 K.

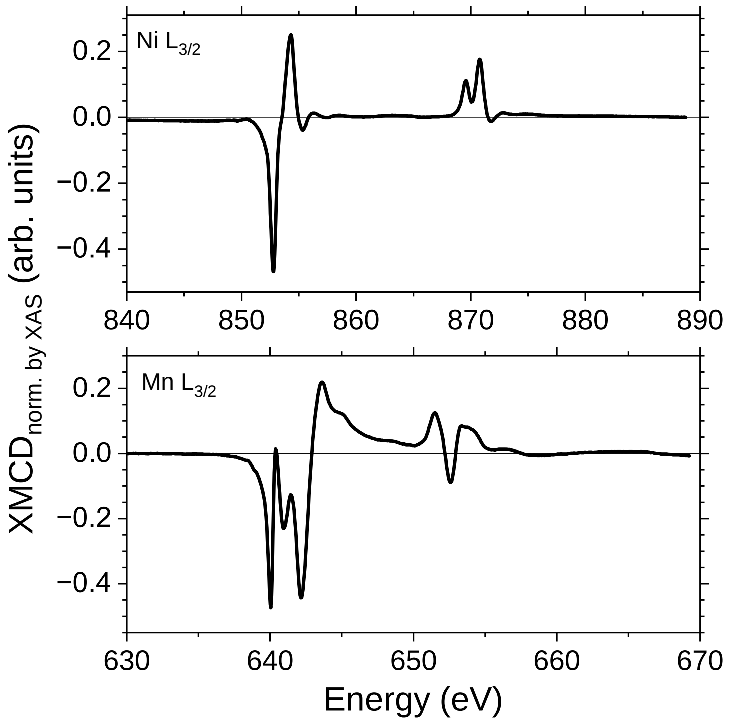

Figure S6: XMCD for a $(3,3)_{10}$ superlattice on NGO(001) recorded at the Ni $L_{3,2}$ (top) and Mn $L_{3,2}$ (bottom) absorption edges at an applied magnetic field of 6 T and a temperature of 20 K.

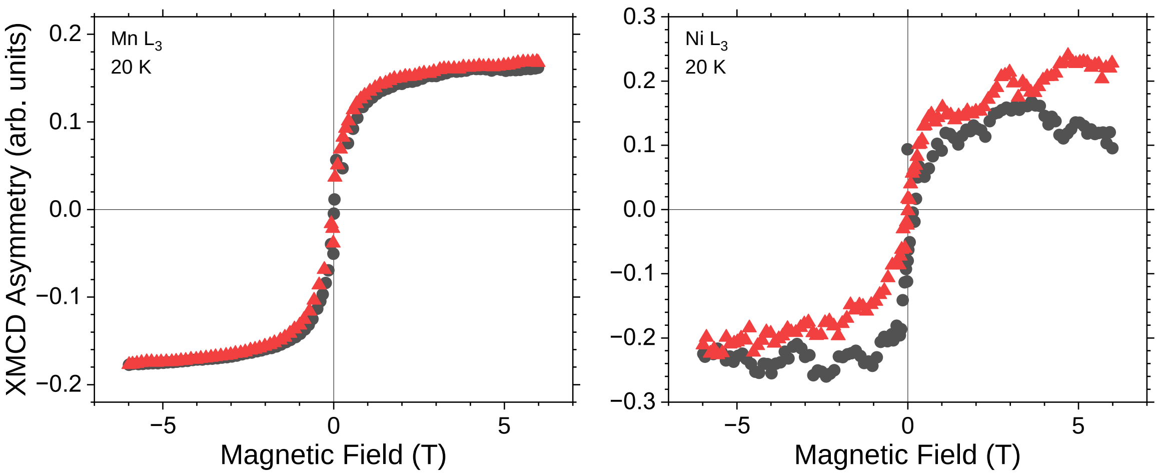

Figure S7: XMCD asymmetry loops for a $(3,3)_{10}$ superlattice on STO(001) recorded at the Mn L_3 (left) and Ni L_3 (right) absorption edges at a temperature of 20 K.

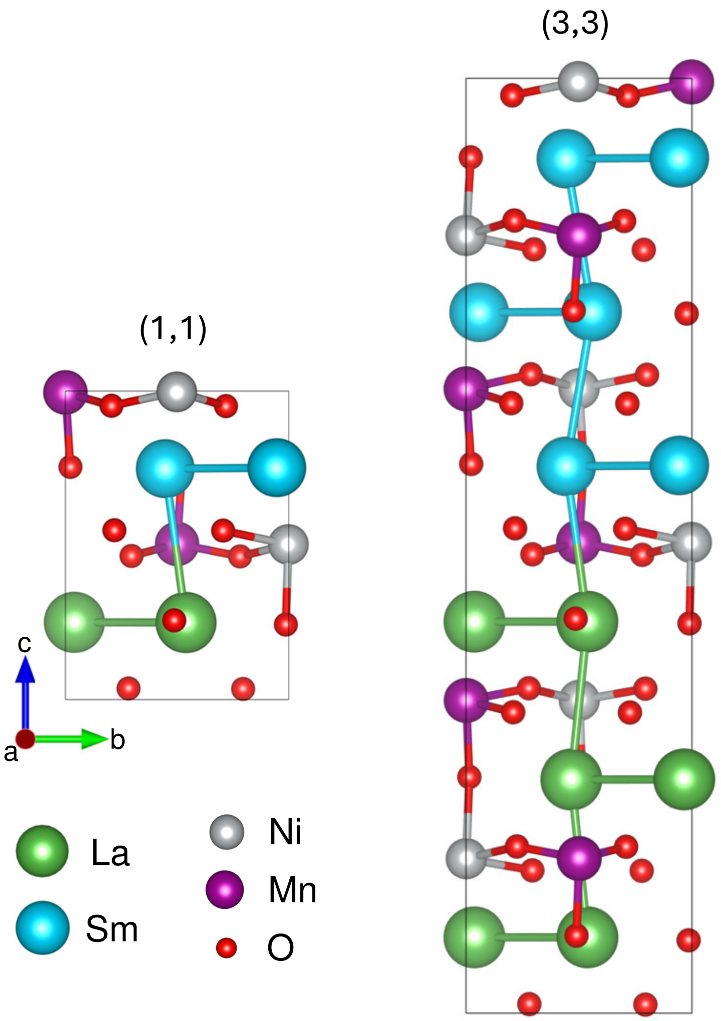

Figure S8: Structures of the DFT-simulated (1,1) and (3,3) cells.

Table S1: Distances along (010) between A-ions within a slab of LNMO or SNMO. The numbers are extracted from the DFT-calculated structure and from STEM images recorded in five different locations. The errors correspond to the standard deviations.

Data	Sm-Sm (pm)	La-La (pm)	Sm-Sm/La-La
DFT	62.7	39.3	1.60
STEM 1	42.6 ± 6.1	26.5 ± 7.0	1.61 ± 0.15
STEM 2	42.8 ± 5.7	24.0 ± 6.5	1.78 ± 0.16
STEM 3	41.9 ± 6.8	23.5 ± 6.0	1.78 ± 0.16
STEM 4	40.4 ± 7.4	22.9 ± 6.9	1.76 ± 0.22
STEM 5	47.0 ± 7.7	29.2 ± 7.2	1.61 ± 0.14
STEM Avg.	42.9 ± 6.7	25.2 ± 6.7	1.71 ± 0.17

The electrically polarized structure in LNMO/SNMO superlattices appears as a secondary order as soon as the octahedral tilts set in. Thus, the corresponding phase transition would coincide exactly with the structural (anteferrodistortive) phase transition involving the octahedral tilts. An accurate calculation of the structural phase transition temperature T_s is challenging. However, one can give a physically sound estimate of it based on the energy difference ΔE between the corresponding high- and low-symmetry phases.[1] Using this approach, we compute the T_s below which octahedral tilts develop in LNMO/SNMO superlattices using the following expression:

$$\Delta E = |E_c - E_{\text{orth}}| = \alpha k_B T_s \quad \text{Eq. (1)}$$

where E_c and E_{orth} are the DFT-calculated energies of the cubic-like and orthorhombic phases (see Methods for more details); k_B is the Boltzmann constant, and α is a proportionality constant. We determine the value of α through $\alpha = \frac{\Delta E}{k_B T_c}$, with $\Delta E = 0.6$ eV/f.u., as computed using DFT for the energy difference between the cubic and tetragonal I4/mcm phases (with octahedral tilts) of $CaTiO_3$ [2], and the experimental T_s value for such transition (T_s=1635 K) [3], see also Table S2. We obtain $\alpha = 4.26$ for a 5 atom cell of $CaTiO_3$. For the (1,1) superlattice, which we simulated using a 20 atom cell, we then use $\alpha = 4 \cdot 4.26 = 17.034$, while for the (3,3) superlattice, which is simulated using 60 atom cell, we use $\alpha = 12 \cdot 4.26 = 51.102$. Accordingly, we predict $T_s \approx$ 1100 and 1500 K for the (1,1) and (3,3) superlattices, respectively, which are significantly above room temperature.

Table S2: DFT-computed values for the energy of the cubic-like E_c and orthorhombic E_{ortho} phases, corresponding energy difference ΔE, proportionality constant α and predicted T_s for the development of rotations for a (1,1) and (3,3) LNMO/SNMO superlattices.

	(1,1)	(3,3)
E_c (eV)	-157.59247	-470.98028
E_{ortho} (eV)	-159.19280	-477.63408
ΔE (eV)	1.60033	6.6538
α (eV)	17.034	51.102
T_s (K)	1090	1510

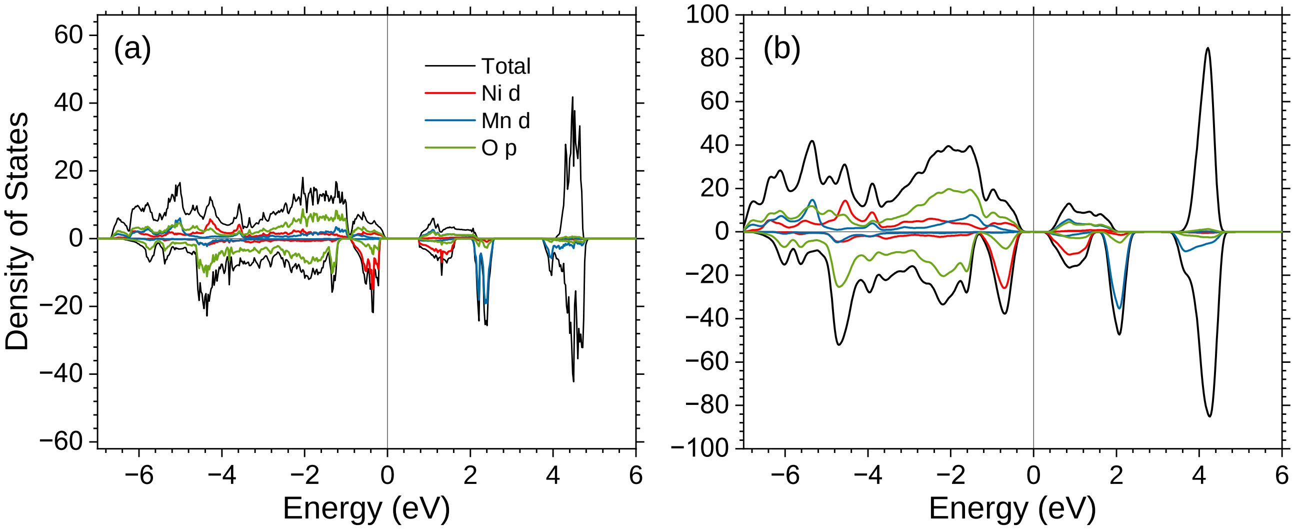

Figure S9: Electronic density of states for (a) (1,1) and (b) (3,3) LNMO/SNMO superlattices.

References

[1] J. C. Wojdeł, J. Íñiguez, *Phys. Rev. B* **2014**, *90* 014105.

[2] P. Chen, M. N. Grisolia, H. J. Zhao, O. E. González-Vázquez, L. Bellaiche, M. Bibes, B.-G. Liu, J. Íñiguez, *Phys. Rev. B* **2018**, *97* 024113.

[3] M. Yashima, R. Ali, *Solid State Ionics* **2009**, *180*, 2 120.
